# Prevalence of Undernutrition Among Tribal Lactating Mothers in Andhra Pradesh, India

**DOI:** 10.7759/cureus.48190

**Published:** 2023-11-02

**Authors:** Khyati Garg, Venkatashiva Reddy B, Rakesh Kakkar, Rajeev Aravindakshan, Arti Gupta

**Affiliations:** 1 Community and Family Medicine, All India Institute of Medical Sciences Mangalagiri, Mangalagiri, IND; 2 Community and Family Medicine, All India Institute of Medical Sciences Bathinda, Bathinda, IND

**Keywords:** malnutrition, tribe, lactating, mother, undernutrition

## Abstract

Background

In Andhra Pradesh, India, tribal communities face unique nutritional challenges due to limited access to healthcare and a predominantly plant-based diet. Maternal undernutrition is a significant concern, impacting the well-being of both mothers and their offspring. This study focuses on assessing the prevalence of undernutrition among tribal mothers in Andhra Pradesh using the BMI-for-age criterion.

Objectives

The aim of this study was to comprehensively evaluate the prevalence of undernutrition among tribal mothers, explore associations with demographic factors, and assess the impact of a nutritional intervention program. The ultimate goal was to contribute to targeted interventions and policies for improving the health and well-being of these communities.

Materials and methods

A cohort study was conducted in the Guntur district of Andhra Pradesh, involving 340 lactating mothers and their infants. Data collection and anthropometric measurements were performed.

Results

The study found that 67 (19.71%) of tribal mothers were underweight at baseline. There were statistically significant associations with the Yenadi tribe, low educational status of the mother, and history of lower segment Caesarean section with a high prevalence of being underweight. No significant associations with age, occupation, or socioeconomic status were observed. Undernutrition was more common among mothers with older children and was associated with specific obstetric factors.

Conclusion

While the prevalence of undernutrition is lower than in some previous studies, it remains a critical concern, particularly within disadvantaged communities. These undernourished mothers face health risks, including anemia. Urgent policy interventions and nutritional programs are needed to address this issue and enhance the well-being of tribal communities in Andhra Pradesh.

## Introduction

Nutrition is an indispensable determinant of overall health and well-being, profoundly impacting the physical and cognitive development of individuals, with particular emphasis on women [1]. Maternal nutrition plays an unequivocal role, not only in the health of mothers but also in the holistic well-being of their offspring [2]. Within the culturally diverse and vibrant state of Andhra Pradesh, India, resides a significant tribal population grappling with unique challenges, chiefly stemming from limited access to adequate nutrition and healthcare services [3].

India is home to approximately 104 million tribal individuals, constituting 8.6% of the nation's populace, dispersed across 705 distinct tribes [4]. Regrettably, these tribal communities have endured marginalization across geographical, socioeconomic, political, and societal dimensions, leading to a national consciousness that often overlooks their plight [4]. Health and healthcare within tribal territories persist as unresolved challenges, and these populations continue to depend primarily on agriculture and forest resources for their livelihoods. Their lifestyles, dietary practices, and overall living patterns retain a significant degree of homogeneity. Indigenous foods are still integral to their sustenance, with a predominantly plant-based diet marked by notable anti-nutritional factors that hinder nutrient absorption and bioavailability. Due to financial constraints, even non-vegetarian tribal populations find it arduous to include animal products in their diets, despite the fact that animal-source foods are consumed in limited quantities [5].

Undernutrition is a pressing public health concern, characterized by inadequate nutrient intake, resulting in growth stunting, compromised immune function, and a spectrum of health complications [6]. Among the diverse methodologies for evaluating undernutrition, the BMI-for-age criterion stands out as an invaluable indicator for assessing the nutritional status of individuals, especially women of childbearing age. It furnishes critical insights into the prevalence of undernutrition among this demographic, making it a pivotal measure for maternal health [7].

Tribal communities in Andhra Pradesh often encounter disparities in healthcare and nutrition, significantly impacting the health and well-being of mothers and children within these communities [8]. Despite ongoing efforts at the national and regional levels to address these disparities, there remains a substantial research gap in understanding the precise prevalence of undernutrition, as determined by the BMI-for-age criterion, among tribal mothers in the state [9].

This research project embarks on a mission to comprehensively assess the prevalence of undernutrition among the women in tribal areas of Andhra Pradesh. The overarching objective is to gain a deep understanding of the nutritional challenges faced by these tribal mothers, ultimately contributing to the formulation of targeted interventions and policies aimed at improving their health and well-being. The journey involves evaluating undernutrition using the BMI-for-age criteria, exploring the relationships between undernutrition and various demographic factors such as age, tribe, occupation, educational status, and socioeconomic standing among tribal mothers [10]. The study promises to provide valuable insights and recommendations that can guide future interventions and policies, ultimately fostering the improved health of tribal communities in Andhra Pradesh.

## Materials and methods

The present study is a data analysis of baseline data from a community-based cohort study conducted in the Guntur district of Andhra Pradesh, India, from June 2021 to April 2022. This study focused on lactating mothers, along with their infants residing within the community. The path to map out all the inhabited areas within the Guntur district of Andhra Pradesh where women from scheduled tribes with at least one child under the age of one year could be found was pursued. In this district, there were a total of 23 Integrated Child Development Services (ICDS) projects. Participation was open to individuals meeting specific criteria: having a residence within the study area for at least six months, being present in the selected household during data collection, belonging to a tribe from Andhra Pradesh, and providing consent to partake in this survey. However, mothers exhibiting symptoms suggestive of acute infection and those withholding their consent were not included.

The sample size took into account an estimated incidence with 15% precision, a 95% confidence level, a design effect of two, and a total of 10 clusters. This led to a cohort size of 340 lactating mothers with infants, each cluster composed of 34 lactating mothers.

To identify the study population, all areas inhabited by scheduled tribes' (ST) lactating mothers with children under one year old in the Guntur district were enumerated using a list received from ICDS, Guntur, Andhra Pradesh. The district comprised 23 ICDS project sites, which were conveniently categorized into urban and rural areas, both served by Anganwadi centers. Urban and rural areas with a minimum of 50 eligible mothers were shortlisted. Five urban and five rural clusters were randomly chosen from the list. The urban clusters were named Guntur, Tenali, Mangalagiri, Amruthalur, and Repalle. The rural clusters comprised Bollapalli, Chilkapuripeta, Amaravati, Prathipadu, and Pedakakani in the Guntur district.

A list of eligible mothers within each selected cluster was compiled, and from this list, 34 eligible mothers were chosen at random. Data collection in both urban and rural areas was conducted by a team consisting of two field workers and one laboratory technician.

A comprehensive house-to-house survey was carried out in the selected clusters, ensuring that all eligible lactating mothers with infants present in these households were considered for participation. Before conducting interviews, the investigators took a moment to introduce themselves to the lactating mothers, providing them with a patient information sheet in the local language, which kindly explained the study's objectives, procedures, and the rights of the participants. Written informed consent in the local language was obtained from the lactating mothers. For not literate women, the patient information sheet and informed consent were explained by investigators in the language the patient comprehended. The informed consent forms were signed by two witnesses from the participant's community to maintain the integrity of consent. Subsequently, interviews were conducted following the scheduled protocol. Following the interviews, anthropometric measurements were performed for the lactating mothers. This process was preferably conducted at the participant's residence or at the local Anganwadi center, with a preference for daylight settings.

To assess undernutrition in adults, the Body Mass Index (BMI) was used, calculated by dividing weight in kilograms by the square of standing height in meters (BMI = weight in kg / (height in cm)²). Common BMI cutoff points were thoughtfully applied to define various levels of malnutrition in postnatal mothers. Operationally, underweight among mothers was defined as BMI < 18.5 kg/m², using the WHO standards. For socioeconomic status classification, the BG Prasad Socioeconomic Status Scale 2022 was used.

Data entry was proficiently executed using Microsoft Excel 2010 (Microsoft Corporation, Redmond, Washington), while the subsequent quantitative data analysis was conducted utilizing IBM SPSS Statistics for Windows, Version 28 (Released 2022; IBM Corp., Armonk, New York), compatible with Windows. This analysis was comprehensive, encompassing the calculation of frequencies and means, and employing statistical tests for significance. A 95% confidence interval was calculated using Microsoft Excel 2010. Both univariate and multivariate analyses were done to account for potential confounding variables.

Ethical clearance was granted by the Ethics Committee of All India Institute of Medical Sciences Mangalagiri, with the reference number AIIMS/MG/IEC/2021-22/105. Informed written consent was taken before enrolling the mothers in this study.

## Results

A total of 340 mothers and children participated in this study. At the baseline, 67 (19.71%) of mothers were identified as undernourished based on the BMI criteria, with approximately 4-5% classified as severely or moderately underweight (Table 1).

**Table 1:** Prevalence of undernutrition among mothers using BMI criterion at baseline (n=340) The data have been presented as n, prevalence in %, and 95% confidence interval.

BMI (kg/m^2^)	Variable	n	Prevalence (%) (95% CI)
<18.5	Undernutrition present	67	19.71 (15.83 to 24.26)
Grading of nutritional status			
<16.0	Severe	12	3.53 (2.03-6.07)
16.0–16.9	Moderate	17	5 (3.14-7.86)
17.0–18.4	At risk	38	11.18 (8.25-14.97)
18.5–24.9	Normal	193	56.76 (51.45-61.93)
25.0–29.9	Overweight	51	15 (11.6-19.19)
>30	Obese	29	8.53 (6-11.98)

The distribution of undernutrition prevalence varied significantly by tribe, with 22 (32.8%) of the mothers from the Lambadi/Sugali tribe and 28 (41.8%) from Yenadi exhibiting undernutrition at baseline (Table 2).

**Table 2:** Distribution of prevalence of undernutrition among mothers by their tribe at baseline (n=340). The data have been presented as n, %. *p<0.05 is considered significant.

Category		Undernutrition baseline							
		Present		Absent		Total		Chi-square	p-value
		n	%	n	%	n	%		
Tribe name	Chenchu	3	4.50%	31	11.40%	34	10.00%	16.93	.002*
	Yerukula	11	16.40%	81	29.70%	92	27.10%		
	Lambadi/Sugali	22	32.80%	83	30.40%	105	30.90%		
	Yenadi	28	41.80%	56	20.50%	84	24.70%		
	Others	3	4.50%	22	8.10%	25	7.40%		

While no significant associations were found between maternal age, occupation, and educational status, the prevalence of undernutrition significantly correlated with education, with 56 (83.58%) of undernourished mothers being non-working/housewives and 26 (38.81%) being illiterate at baseline. Socioeconomic status also showed no significant association; however, undernutrition was most prevalent among mothers in the lower class, with 30 (44.78%) cases using the BG Prasad Socioeconomic Status Scale 2022 (Table 3).

**Table 3:** Distribution of prevalence of undernutrition among mothers by age, occupation education, and socioeconomic status at baseline (n=340) The data have been presented as n, %. *p<0.05 is considered significant.

Variable		Undernutrition among mothers at baseline					Fischer exact test	p-value
		Present		Absent		Total		
		n	%	n	%			
Age in years	<21	15	22.39	50	18	65	2.521	0.284
	21 to 30	51	76.12	207	76	258		
	>30	1	1.49	16	6	17		
Occupation	Housewife/not working	56	83.58	232	85	288	0.929	0.819
	Agricultural labour	10	14.93	37	14	47		
	Business/ volunteers/self	0	-	2	1	2		
	Job	1	1.49	2	1	3		
Education	Illiterate	26	38.81	116	42	142	18.727	.002*
	Primary level	18	26.87	46	17	64		
	Middle-class level	13	19.4	20	7	33		
	Secondary level	6	8.96	36	13	42		
	Higher Secondary level	1	1.49	31	11	32		
	Graduate or above	3	4.48	24	9	27		
Socioeconomic status (BG Prasad Socioeconomic Scale 2022)	Upper	1	1.49	0	0	1	5.715	0.221
	Upper middle	1	1.49	12	4	13		
	Middle	12	17.91	41	15	53		
	Lower middle	23	34.33	89	33	112		
	Lower	30	44.78	131	48	161		
Total		67	100	273	100	340		

Husband's occupation and education were not significantly related to maternal undernutrition; however, 46 (68.66%) of undernourished mothers had husbands in the middle-class category, and 26 (38.81%) had husbands who were "illiterate" at baseline. The age of the last child in months showed no significant associations, but undernutrition was more prominent among mothers with children older than six years, affecting around 43 (64.18%) (Table 4).

**Table 4:** Distribution of prevalence of undernutrition among mothers by husband education, occupation, and age of children at baseline (n=340) The data have been presented as n, %. *p<0.05 is considered significant.

Variable		Undernutrition among mothers at baseline					Chi-square	p-value
		Present		Absent		Total		
		N	%	n	%			
Husband occupation	Cultivator	5	7.46	17	6	22	4.435	0.35
	Laborer	46	68.66	183	67	229		
	Business/volunteers/self	8	11.94	52	19	60		
	Job	8	11.94	18	7	26		
Husband education (n=337)	Illiterate	26	38.81	108	40	134	5.909	0.315
	Primary level	16	23.88	39	14	55		
	Middle-class level	2	2.99	18	7	20		
	Secondary level	10	14.93	32	12	42		
	Higher secondary level	4	5.97	19	7	23		
	Graduate or above	9	13.43	54	20	63		
Age of child in months	<6	24	35.82	103	38	127	0.084	0.772
	≥6	43	64.18	170	62	213		
Total		67	100	273	100	340		

The prevalence of undernutrition among mothers with Gravida < 3 was 47 (70.17%), Parity ≥ 3 was 51 (76.12%), no male child was 24 (35.82%), no female child was 25 (37.31%), and those who had undergone LSCS was 56 (83.58%) (Table 5). The sex of the child did not significantly affect the prevalence of anemia, but undernutrition among mothers who underwent LSCS in previous pregnancies was statistically significant, with a p-value < 0.001.

**Table 5:** Distribution of prevalence of undernutrition among mothers by obstetrics parameter at baseline (n=340). The data have been presented as n, %. ^p<0.05 is considered significant. *Reference category.

Variable	Category	Undernutrition baseline						
		Present		Absent		Total	Chi-square	p-value
		n	%	n	%			
Number of times pregnant in the past	≥3*	20	29.85	102	37.36	122	1.319	0.251
	<3	47	70.15	171	62.64	218		
Parity	≥3*	51	76.12	207	75.82	258	0.003	0.96
	<3	16	23.88	66	24.18	82		
Male child in the family	Absent*	24	35.82	110	40.29	134	0.451	0.502
	Present	43	64.18	163	59.71	206		
Female child in the family	Absent*	25	37.31	78	28.57	103	1.947	0.163
	Present	42	62.69	195	71.43	237		
Previous lower segment caesarean section (LSCS) done	Absent*	11	16.42	127	46.52	138	20.215	<0.001^
	Present	56	83.58	146	53.48	202		
Total		67	100	273	100	340		

## Discussion

Undernutrition is most often present in disadvantaged communities. Pregnant women and lactating mothers, among the most vulnerable populations, reflect the overall health of the population through their nutritional state. Undernutrition is one of the main risk factors for anemia. In a baseline survey, 67 (19.71%) of mothers were undernourished, using a BMI of less than 18.5 kg/m^2^ as the criteria. Of these, 17 (5%) had a BMI of less than 16 to 16.9, and 12 (3.5%) had a BMI of less than 16. The World Health Organization categorizes adult women with a BMI of less than 18.5 kg/m^2^ as having a medium prevalence of undernutrition, indicating a poor situation. Thinness, defined as a BMI of less than 17.0 kg/m^2^, is linked to an increase in illness among adults. A BMI of less than 16.0 kg/m^2^ is considered an extreme limit and has been associated with an increased risk of illness, poor physical performance, lethargy, and even mortality [7].

Globally, 10% of women aged 20 to 49 are underweight [11]. According to the 2022 Global Nutrition Report, 22.7% of India's women over 18 years of age are undernourished [12]. In Andhra Pradesh, 14.8% of women (aged 15-49 years) had a BMI of less than 18.5 kg/m^2^, as reported by the National Family Health Survey (NFHS 5) [13]. In 2017, a study reported that 10.1% of 540 Chakhesang women of the tribe in Nagaland, North-East India, were undernourished [14].

A higher overall prevalence of undernutrition among women was reported in other studies. In the years 2007-08, the National Nutrition Monitoring Bureau of India conducted research on the nutritional status of adults in Indian tribes, finding that 49% of the women were undernourished [15]. From 2011 to 2013, a cross-sectional study involving 1,090 females aged 20-60 years from nine major tribes: Santals, Oraons, and Koras in West Bengal; Santals, Bhumijs, and Bathudis in Odisha; and Dhodias, Kuknas, and Chaudharis in Gujarat found that, overall, 516 (47.4%) of the females were undernourished [11]. In 2015, a survey from 21 tribal villages in West Bengal documented that nearly half (49.4%) of the mothers were underweight, with a BMI of less than 18.5 kg/m^2^ [16]. Over 40% of lactating mothers were undernourished using BMI criteria among the KOL tribe in Chitrakoot, Uttar Pradesh, in a cross-sectional study in 2021 [17].

The higher prevalence of undernutrition among women in the above studies could be attributed to differences in the age groups studied, geographic locations, and poor health indicators in the study areas. Despite this, the present study documented a lower burden of undernutrition among mothers. Alarmingly, among the undernourished mothers, half were at risk of morbidity and even mortality. Although women's undernutrition is a severe health issue, particularly among disadvantaged and vulnerable groups, practical policies in nutritional intervention programs have not been sufficiently emphasized. Focusing solely on the Guntur district of Andhra Pradesh may limit the generalizability of the findings to other tribal regions in India, which might exhibit variations in nutritional challenges.

By thoroughly investigating associations with a wide range of demographic variables, this research offers a multifaceted view of maternal undernutrition in tribal communities. Despite a lower prevalence compared to previous studies, our research underscores the persistent challenge of maternal undernutrition in tribal communities. It emphasizes the urgency for policy interventions, particularly for disadvantaged groups, and highlights the potential health risks faced by undernourished mothers. It is important to recognize the regional specificity and sample size limitations when interpreting the findings. Acknowledging these limitations, our study's strengths lie in its comprehensive approach, ethical considerations, and the depth of insights it provides into the intricate issue of maternal undernutrition among tribal mothers in Andhra Pradesh.

## Conclusions

Our research reveals a concerning 19.71% prevalence of undernutrition among tribal mothers in the Guntur district of Andhra Pradesh. This rate is notably high, encompassing both severe and moderate cases of underweight. Undernutrition among mothers is significantly linked to the Yenadi tribe and low education status. It is more prevalent among mothers with older children and tied to certain obstetric factors. Although this prevalence is less severe than that observed in some previous studies, it remains a vital concern for disadvantaged communities. These undernourished mothers face health risks, including anemia. Urgent policy and nutrition programs are necessary to address this issue and improve community health.

## References

[1] Otunchieva A, Smanalieva J, Ploeger A (2022). Dietary quality of women of reproductive age in low-income settings: a cross-sectional study in Kyrgyzstan. Nutrients.

[2] Dunneram Y, Jeewon R (2015). Healthy diet and nutrition education program among women of reproductive age: a necessity of multilevel strategies or community responsibility. Health Promot Perspect.

[3] Laxmaiah A, Kodavanti MR, Rachakulla HK, Arlappa N, Venkaiah K, Brahmam GNV (2007). Diet and nutritional status of tribal population in ITDA project areas of Khammam district, Andhra Pradesh. J Hum Ecol.

[4] (2021). Report of the Expert Committee on Tribal Health, tribal health in India, bridging the gap and a roadmap for the future, policy brief. https://nhm.gov.in/index1.php?lang=1&level=1&sublinkid=1110&lid=630.

[5] Gupta A, Kollimarla M, Reddy B V, Noorani Shaik Y, Kakkar R, Aravindakshan R (2022). Exploring unknown predictors of maternal anemia among tribal lactating mothers, Andhra Pradesh, India: a prospective cohort study. Int J Womens Health.

[6] (2022). Malnutrition in women. https://www.who.int/data/nutrition/nlis/info/malnutrition-in-women.

[7] (2020). BMI classification. World Health Organization. http://apps.who.int/bmi/index.jsp?introPage=intro_3.html.

[8] Babu KS (2012). Socio-Economic and Health Conditions of Some Major Tribes in Andhra Pradesh. History Research J.

[9] (2021). Ministry of Tribal Affairs: Government of India. http://tribal.nic.in.

[10] Gupta A, Shaik YN, Kakkar R, Aravindakshan R, Pentapati SS, Reddy BV (2022). Effectiveness of weekly local mothers' kitchen recipe talks to improve dietary iron intake among tribes of Andhra Pradesh, India: a prospective cohort study. J Family Med Prim Care.

[11] Kshatriya GK, Acharya SK (2016). Gender disparities in the prevalence of undernutrition and the higher risk among the young women of Indian tribes. PLoS One.

[12] (2023). 2022 Global Nutrition Profile. https://globalnutritionreport.org/resources/nutrition-profiles/asia/southern-asia/india/.

[13] A National Family Health Survey-5, 2019-20, International Institute for Population Science. http://rchiips.org/nfhs/NFHS-5_FCTS/Andhra_Pradesh.pdf.

[14] Longvah T, Khutsoh B, Meshram II, Krishna S, Kodali V, Roy P, Kuhnlein HV (2017). Mother and child nutrition among the Chakhesang tribe in the state of Nagaland, North-East India. Matern Child Nutr.

[15] National Nutrition Monitoring Bureau (2009). National Nutrition Monitoring Bureau. NNMB Technical Report No. 25-Diet and Nutritional Status of Tribal Population and Prevalence of Hypertension among Adults—Report on Second Repeat Survey. NNMB Technical Report No. 25 - Diet and Nutritional Status of Tribal Population and Prevalence of Hypertension Among Adults—Report on Second Repeat Survey.

[16] Stiller CK, Golembiewski SK, Golembiewski M, Mondal S, Biesalski HK, Scherbaum V (2020). Maternal nutritional status and child feeding practices: a retrospective study in Santal communities, Birbhum District, West Bengal, India. Int Breastfeed J.

[17] Yadav S, Ahmad S, Chaudhary N, Kumar M, Gahlot A (2021). Dietary behaviour and anthropometric parameters across the spectrum of pregnant and lactating mother, infant, young children, adolescent girls and reproductive age group females: an assessment of undernutrition among KOL tribe. J Family Med Prim Care.

